# Lung cancer and peritoneal carcinomatosis

**DOI:** 10.3892/ol.2013.1468

**Published:** 2013-07-15

**Authors:** MARÍA SERENO, ISABEL RODRÍGUEZ-ESTEBAN, CÉSAR GÓMEZ-RAPOSO, MARÍA MERINO, MIRIAM LÓPEZ-GÓMEZ, FRANCISCO ZAMBRANA, ENRIQUE CASADO

**Affiliations:** 1 Department of Oncology, Infanta Sofía University Hospital, San Sebastián de los Reyes, Madrid 28708, Spain; 2 Department of Pathology, Infanta Sofía University Hospital, San Sebastián de los Reyes, Madrid 28708, Spain

**Keywords:** lung cancer, peritoneal carcinomatosis, intestinal perforation

## Abstract

Lung cancer is currently one of the most common malignancies in the world and peritoneal involvement is rare in these types of tumors. Clinical manifestations of these metastases are also uncommon and include intestinal perforation and obstruction. The present study reviewed certain aspects of the complication of peritoneal involvement and illustrated it with four cases of patients that were diagnosed with primary lung carcinoma and secondary peritoneal carcinomatosis (PC). The outcome of these patients is poor and they rarely respond to chemotherapy. Surgery is successful in the majority of cases.

## Introduction

Lung cancer is currently one of the most common malignancies in the world. In the year 2002, 1.35 million new cases were diagnosed, which represented 12.4% of all the newly-diagnosed cancers worldwide. Metastatic disease is observed in ~40% of patients with lung cancer, with the most common sites of metastasis being the bone, liver, brain and adrenal glands.

Peritoneal carcinomatosis (PC) is defined as the progression of the primary cancer to the peritoneum. Although the condition may be diagnosed by surgical procedures, including a laparoscopic peritoneal biopsy, the majority of patients are diagnosed with PC using imaging techniques, such as computed tomography (CT), or by the presence of malignant cells in the ascitic fluid.

PC is a rare clinical event in lung cancer patients, with autopsy results showing an incidence of 2.7–16%. In a study by McNeill *et al*(1), peritoneal and small bowel metastases were observed in 46 of 431 patients with primary lung cancer who underwent autopsies during an 11-year period. These patients had an average of 4.8 metastatic sites. Small bowel metastases were present in 12 of 31 (39.0%) patients with large cell carcinoma, 13 of 108 (12.3%) patients with adenocarcinoma, six of 73 (8.0%) patients with small cell carcinoma, 15 of 199 (7.5%) patients with squamous cell carcinoma and none of the 20 (0.0%) patients with undifferentiated carcinoma (1).

The clinical manifestations of these metastases are rare, with intestinal perforation and obstruction being the most common (2).

A review of the literature identified only clinical case studies. The present study reviewed four cases of patients that were diagnosed with PC secondary to primitive lung carcinoma, in order to describe the characteristics of this rare complication. Written informed consent was obtained from the patients.

## Cases reports

### Case 1

A 64-year-old male, who had been a heavy smoker for 30 years (>60 pack-years), was studied in the Department of Pulmunology (Infanta Sofía University Hospital San Sebastián de los Reyes, Madrid, Spain) due to a progressive dyspnea. The chest X-ray revealed a massive left pleural effusion. A thoracocentesis was performed for evacuation, which resulted in a significant symptomatic improvement. The bronchoscopy revealed an extrinsic compression secondary to the pleural effusion. A cytological examination showed positive staining for malignant cells, which is consistent with trefoil factor (TFF)-1 adenocarcinoma metastases. A hydropneumothorax was observed on a body CT as a complication of the biopsy, which used the fine-needle aspiration procedure. The chest CT revealed pleural effusion and pathological mediastinal nodes. A chest tube was required for the drainage of the pleural effusion. An epidermal growth factor receptor (EGFR) activating mutation was detected in the cytological examination (deletion in exon 19).

**Figure 1:**
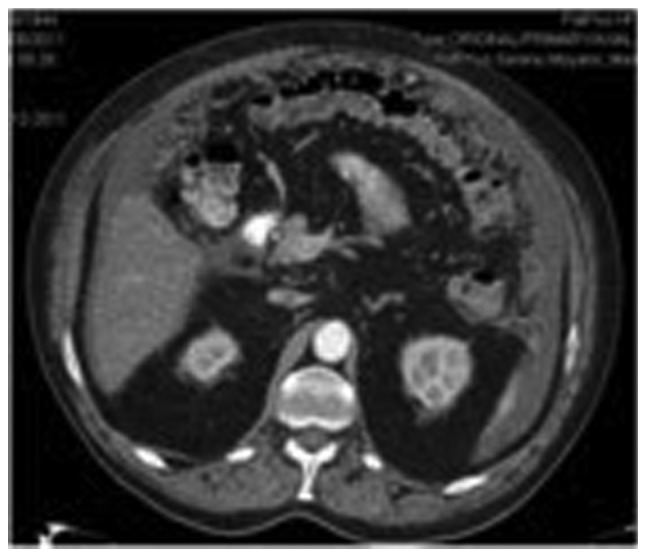
A marked thickness of the omentum and of the mesentery with ascites.

At the time that the patient presented to the hospital, anti-EGFR treatment in the first-line setting for patients with EGFR-activating mutations was not approved. Therefore, the patient was treated using chemotherapy. A biweekly regimen was administered based on a combination of cisplatin (50 mg/m^2^) and docetaxel (50 mg/m^2^). The patient underwent eight courses of chemotherapy with a partial response. Subsequent to terminating the chemotherapy, the patient presented with an asymptomatic pulmonary embolism in a follow-up CT scan and anticoagulant treatment was consequently administered. Progression of pleural effusion and an increased number of mediastinal nodes was identified 10 months later in a routine chest CT. A new regimen treatment with erlotinib was administered, resulting in a stable disease state for one year. In a body scan, a pleural progression was described. In the abdomen, a marked thickness of the epiplon and the mesentery was observed with ascitis (Fig. 1). All these findings were suggestive of PC. A guided CT biopsy was obtained from the peritoneum that confirmed lung adenocarcinoma metastasis. A new EGFR analysis was performed to determine the acquired resistant mutations. A double mutation was detected consisting of an activating mutation (exon 19 deletion) and an acquired resistance mutation (exon 20 T790M).

The patient underwent a third-line treatment and was stable following eight courses of pemetrexed and six of carboplatin and paclitaxel following the previous regimen. A partial response was observed in the last CT.

The patient is no longer undergoing chemotherapy and has not exhibited any symptoms of progression while waiting for a new evaluation.

### Case 2

A 52-year-old female who was a heavy smoker (30/day, for 20 years) with no medical conditions was admitted to the emergency room (ER) due to an intensive and progressive pain in the left shoulder. In the chest X-ray, a pulmonary mass was detected in the left upper lobe. The chest CT demonstrated a 4.9×1.6-cm mass in the left upper lobe, which was consistent with the radiological findings of emphysema. A contralateral 2.5-cm lesion was observed in the right upper lobe and was associated with the pleural thickness in addition to smaller lesions in the two lungs, which were suggestive of metastatic lesions. In the abdomen, several nodules were identified in the greater omentum (the larger lesion was ~1.6 cm in diameter), which were consistent with PC. A fine-needle aspiration puncture of the lesion in the left upper lobe was performed. The pathological findings were consistent with TFF1-positive lung adenocarcinoma and no EGFR activating mutations or echinoderm microtubule-associated protein like 4-anaplastic lymphoma kinase (EML4-ALK) translocations were detected. The patient was administered chemotherapy based on cisplatin (75 mg/m^2^) and pemetrexed (500 mg/m^2^). Following six courses of this regimen, a radiological evaluation was scheduled. A partial response was obtained and maintenance with pemetrexed was initiated. At five months post-treatment, the patient presented with worsening dyspnea and a body scan revealed a pulmonary progression. Therefore, a second-line regimen based on docetaxel was administered. Following nine courses of chemotherapy, a clinical and radiological response was observed. Since asthenia grade I, mild nail toxicity and neuropathy grade I were observed, the chemotherapy was stopped and the patient was administered a maintenance regimen with erlotinib. The patient is currently on treatment and is clinically and radiologically stable.

### Case 3

**Figure 2:**
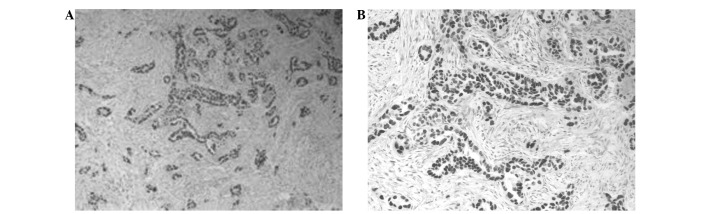
Lung adenocarcinoma biopsy showing positive TFF1 staining. Magnification (A) ×10 and (B) ×20. TFF1, trefoil factor 1.

**Figure 3:**
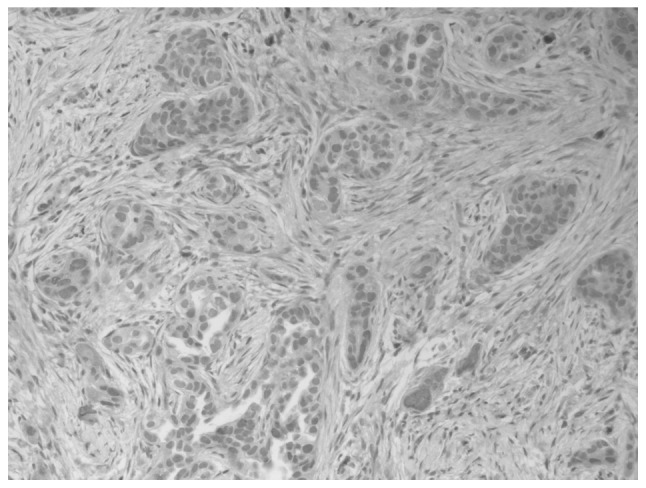
Lung adenocarcinoma biopsy showing positive calretinin staining.

A 63-year-old male was admitted to the ER due to shortness of breath, a cough and chest pain. The X-ray of the chest revealed a right pleural effusion. A diagnostic thoracocentesis was performed and a sample of the pleural effusion was sent for pathological examination. Malignant cells that were positive for TFF1 and carcinoembryonic antigen (CEA) expression were detected, which was suggestive of adenocarcinoma. The CT showed a compressive atelectasis involving the right lower lobe and an extensive pleural effusion. In the abdomen, a malignant 2-cm nodule was observed in the left adrenal gland. A positron emission tomography (PET)-CT scan confirmed the CT findings, which depicted pathological uptakes in the right lower lobe and several uptakes in the right pleural cavity and the adrenal nodule. A pleural biopsy was performed and the pathologist confirmed the diagnosis of adenocarcinoma. However, the immunohistochemical analysis of the pleural tissue was inconsistent with the analysis of the ion pleural effusion sample. The biopsy revealed that the tissue was negative for TFF1 and calretinin, but positive for CEA and cytokeratin (CK)-7 (Figs. 2 and 3). The pleural liquid sample analysis revealed positive results for TFF1, CEA and CK7. The colonoscopy and stomach endoscopy did not indicate any lesions.

The final diagnosis was of pleural metastasis from lung adenocarcinoma. The EGFR analysis did not detect any activating mutations. Therefore, the patient was administered a chemotherapy regimen based on carboplatin-docetaxel-bevacizumab, but presented with grade III [World Health Organization (WHO) classification] hematological toxicity following the first course (1). Consequently, a dose reduction was required. The patient underwent six courses, with a partial response, and continued with bevacizumab as a maintenance treatment. Following three months of treatment, the CT scans showed an increased pleural effusion, pulmonary nodules and several images that were suggestive of PC. A demonstrative biopsy of the peritoneum was performed and the pathological findings were consistent with malignant mesothelioma of epithelioid subtype. The immunophenotype demonstrated positive calretinin staining and was negative for CK7, CK20, prostate specific antigen (PSA), TTF1, p63, CD10 and α-fetoprotein (FP). Ki67 was expressed in ~50% of the cells.

A new line of chemotherapy was administered based on pemetrexed. The patient received three courses, presenting with toxicity grade II. The CT scans showed a stable disease. A pulmonary embolism was detected following the fourth administration and the patient succumbed several days later.

### Case 4

A 67-year-old male was admitted to the ER with breathlessness and chest pain. The patient suffered from diabetes and hyperuricemia, which was kept under control using alopurinol and metformina.

The chest X-ray revealed a large right pleural effusion and the CT showed a compressive atelectasis and a right hilar mass. The pathological analysis of the pleural effusion was consistent with the malignant cells from the immunohistochemical pattern, suggesting a diagnosis of adenocarcinoma (TFF1-positive, CEA-positive, CK7-positive, CK20-negative and calretinin-negative). The pleural biopsy described a pleura that was infiltrated by a primary lung adenocarcinoma. The patient was diagnosed with stage IVB lung adenocarcinoma due to the positive pleural effusion. A chemotherapy regimen based on carboplatin, paclitaxel and bevacizumab was initiated. A partial response was observed, but the treatment had to be stopped due to hematological toxicity. EGFR mutations were identified involving a mutation in exon 18, G719S. The patient underwent treatment with erlotinib, which was well tolerated. Erlotinib was discontinued due to a pleural and mediastinal progression and a new chemotherapy regimen based on pemetrexed was administered. Following nine courses of chemotherapy resulting in stabilization, the patient suffered a new progression in the peritoneum and liver metastasis. The patient was admitted to the ER with a bowel obstruction that was resolved with supportive treatment. Docetaxel was administered every two weeks, which improved the clinical status. The radiological evaluation revealed a stable disease.

## Discussion

Lung carcinoma is the leading cause of cancer-related mortality and ~50% of cases exhibit distal metastasis at the time of diagnosis (1). The preferential sites of extrapulmonary metastasis are the lymph nodes, liver, adrenal gland, bone and brain.

Peritoneal metastasis of primary lung carcinoma is considered to be very rare, although it is identified in 2.7–16% of all lung cancer patients (3). Clinical studies concerning this distant metastasis are rare. Satoh *et al* reviewed 1,041 lung cancer patients over a 26-year period and 8 cases (0.77%) developed clinical PC. In the present study, the most common histological type of lung cancer that was associated with PC was adenocarcinoma, accounting for >80% of the cases with PC, and unlike the study by Satoh *et al*, no patient with large cell lung cancer was diagnosed with PC, which was relatively rare in patients with small cell and squamous cell lung cancer (4). In a previous study, the median survival from the diagnosis of PC was approximately two months and half of the patients developed liver or abdominal lymph node metastases (2).

Occasionally, PC causes a reconsideration of the initial pathological diagnosis and requires the consideration of alternative diagnoses, such as that of mesothelioma. This is a not a rare situation, particularly when the initial diagnosis has been established using cytology. A patient in one of the previously mentioned case studies (3) was initially diagnosed with pleural metastasis from lung adenocarcinoma according to the pleural biopsy and pleural effusion sample: TTF1 and calretinin was negative and CEA and CK7 were positive in the biopsy results. However, in the pleural liquid sample, the TTF1, CEA and CK7 results were positive. This pattern was suggestive of lung adenocarcinoma, and the patient underwent treatment according to this diagnosis. However, when the patient later presented with a peritoneal progression, a new and larger biopsy was taken and the pathological diagnosis was consistent with malignant mesothelioma of epithelioid subtype. The immunophenotype demonstrated positive calretinin staining and was negative for CK7, CK20, PSA, TTF1, P63, CD10 and α-FP. The new diagnosis may have been due to the small size of the previous pleural biopsy, or due to an incomplete pattern of antibodies (5). The distinction between epithelioid mesothelioma and lung adenocarcinoma remains a significant diagnostic challenge for surgical pathologists. Kushitani *et al* demonstrated that for distinguishing between epithelioid mesothelioma and lung adenocarcinoma, the combination of CEA, calretinin and either WT1 or thrombomodulin would form the best panel of immunohistochemical markers (5). Therefore, in a patient with PC and primary lung adenocarcinoma, a possible metastatic pleural mesothelioma must first be ruled out by checking the immunohistochemical pattern of the antibodies that are used in the pathological examination.

In the present study, using a cytological examination, the patient from Case 1 was identified to harbor an EGFR-activating mutation (deletion in exon 19). Currently, there is no published evidence with regard to the frequency of EGFR-activating mutations in patients with lung adenocarcinoma and secondary PC. In a multivariate analysis of the incidence of EGFR mutations, including the presence or absence of brain or bone metastases, Rosell *et al* identified no association between the prognosis and the location of the metastasis (6). In this study, we have presented the first case to be published in the literature that describes a primary lung adenocarcinoma with peritoneal metastasis and an EGFR mutation (deletion in exon 19) (Case 1). In patients with advanced non-small cell lung cancer, activating mutations in the EGFR gene confer hypersensitivity to the tyrosine kinase inhibitors, gefitinib and erlotinib. The patient was first administered erlotinib and maintained a good response for one year, according to the literature data (14 months) (7). The patient then presented with a peritoneal progression and a second biopsy was planned to rule out acquired resistant EGFR mutations. A double mutation was detected, consisting of an activating mutation (exon 19 deletion) and an acquired resistance mutation (exon 20 T790M). Due to these findings, a new chemotherapy regimen was administered, resulting in a stabilization of the peritoneal mass and a considerable improvement in the ascitis and abdominal perimeter. Case 4 also involved an EGFR mutation in exon 18, but the patient responded poorly to anti-EGFR.

Su *et al*(7) have published a lung cancer and PC study in which four patients presented with EGFR mutations and were treated with the EGFR tyrosine kinase inhibitor, gefitinib. Two patients, who responded to gefitinib therapy, demonstrated improved abdominal conditions with gradually diminishing ascites and survived for 203 and 343 days, respectively. Therefore, according to these data, activating EGFR mutations in lung carcinoma, even in cases with peritoneal disease, are considered positive predictors of anti-EGFR therapy (8). With the exception of the EGFR-positive tumors, the majority of lung adenocarcinomas with PC have poor prognoses. Aggressive systemic chemotherapy following the diagnosis of PC in lung cancer patients is not associated with an improved outcome. The reasons for this are the poor performance status of these patients and the possibility of developing undesirable complications, including perforation and obstruction. Due to advances in the improvement of chemotherapy and supportive care for lung cancer and the ability to extend life expectancy, an increasing number of this kind of metastatic tumor may be encountered in the future. Therefore, more attention should be focused on gastrointestinal (GI) metastatic signs, including GI bleeding, epigastric pain, nausea, vomiting, acute abdominal pain, or less commonly, ileus. The factors that affect the development of complications from PC are unknown. The histological type, tumor grade and other biological parameters may play significant roles. Contributing factors are immunosuppression, chemotherapy, radiotherapy and the use of cortisone and other drugs (9).

Certain chemotherapeutic agents, including antiangiogenic drugs have been suggested to contribute to the occurrence of perforation (10).

More effort should be made to improve the management of this clinically rare complication of lung cancer that has a poor prognosis, avoiding critical complications, including perforation and intestinal obstruction (8).
